# Sharing and Safeguarding Pediatric Data

**DOI:** 10.3389/fgene.2022.872586

**Published:** 2022-06-20

**Authors:** Dimitri Patrinos, Bartha Maria Knoppers, David P. Laplante, Noriyeh Rahbari, Ashley Wazana

**Affiliations:** ^1^ Centre of Genomics and Policy, School of Biomedical Sciences, Faculty of Medicine and Health Sciences, McGill University, Montreal, QC, Canada; ^2^ Lady Davis Institute (LDI), Montreal, QC, Canada; ^3^ Centre for Child Development and Mental Health, Jewish General Hospital, Montreal, QC, Canada

**Keywords:** data sharing, pediatric research, research ethics, genomics, policy

## Abstract

Data sharing is key to advancing our understanding of human health and well-being. While issues related to pediatric research warrant strong ethical protections, overly protectionist policies may serve to exclude minors from data sharing initiatives. Pediatric data sharing is critical to scientific research concerning health and well-being, to say nothing of understanding human development generally. For example, large-scale pediatric longitudinal studies, such as those in the DREAM-BIG Consortium, on the influence of prenatal adversity factors on child psychopathology, will provide prevention data and generate future health benefits. Recent initiatives have formulated sound policy to help enable and foster data sharing practices for pediatric research. To help translate these policy initiatives into practice, we discuss how model consent clauses for pediatric research can help address some of the issues and challenges of pediatric data sharing, while enabling data sharing.

## Introduction

Biomedical research has become increasingly data-intensive and collaborative in nature (Rahimzadeh, 2017). The sharing of data, including genomic and health-related data, within the wider research community is key to our understanding of human health and well-being, and serves to foster scientific advancements and their benefits (Knoppers et al., 2014; Lo, 2015). Indeed, data sharing is recognized as both an ethical and scientific imperative (Bauchner et al., 2016; Knoppers et al., 2011; Knoppers et al., 2014).

However, minors are very often excluded from data sharing initiatives, especially where genomic data is involved (Rahimzadeh et al., 2018). The sensitivity of genomic data, consent-related issues, and the uncertain risk of re-identification have all been raised as reasons for excluding minors from data sharing (Rahimzadeh et al., 2020). International research ethics guidelines recognize the unique vulnerability of minors as research participants and the requirement for the implementation of special protections for minors (e.g. Council for International Organizations of Medical Sciences, 2016; World Medical Association, 2013). Overly protectionist policies have been noted to unduly restrict data sharing practices which may benefit minors’ health and well-being (Beauvais et al., 2021). While research ethics guidelines recognize the vulnerability of minors as research participants, they also recognize that minors have unique health needs and that childhood diseases often have distinct etiologies (Canadian Institutes of Health Research et al., 2018; Council for International Organizations of Medical Sciences, 2016). Restricting data sharing can therefore impede research which may benefit both current and future generations of minors and is specific to their pediatric health needs.

Recent calls have been made in the literature for greater pediatric data sharing (Rahimzadeh et al., 2020; Vaske & Haussler, 2019). Nevertheless, the unique vulnerability of minors as research participants has long been recognized, which presents specific challenges in sharing pediatric data (Beauvais et al., 2021; Rahimzadeh et al., 2017). While data sharing is key in accelerating scientific discovery, it poses numerous ethical and legal challenges. For one, sharing data with other researchers entails the potential risk of loss of confidentiality through re-identification (Figueiredo, 2017). The implications for loss of privacy have been argued to be greater when data is being shared (Aleixandre-Benavent et al., 2019). Given that minors generally lack the capacity to provide informed consent, which is instead provided by their parent(s) or legal guardian(s), who may not adequately comprehend and appreciate this risk (Council for International Organizations of Medical Sciences, 2016; Daly, 2020). It could therefore be argued that, in the absence of the cognitive capacity to make the benefit-risk analysis of data sharing, their data should not be shared.

Similarly, arguments predicated upon the minor’s moral “right to an open future” have been used to argue against data sharing. The potential for re-identification, in particular, may infringe upon this moral right, which protects the minor from having certain future rights foreclosed upon (Feinberg, 1980; Millum, 2014). Sharing a minor’s data with other researchers may increase the risk of re-identification, which could then have significant implications for the minor and their future. For these reasons, some authors have argued that parents (or legal guardians) should not be able to provide consent for the sharing of their children’s data (Aleixandre-Benavent et al., 2019). Rather, this choice should be reserved for the minor themselves, when they reach the age of majority (Brothers, 2011; Aleixandre-Benavent et al., 2019)

Accordingly, to address the challenges of pediatric data sharing, in 2018 the interdisciplinary Paediatric Task Team of the Global Alliance for Genomics and Health (GA4GH) - an international consortium that develops standards for the responsible governance of genomic data - developed the Key Implications for Data Sharing (KIDS) framework for pediatric genomics. This framework outlines four policy points for the responsible sharing of pediatric data: 1) the involvement of minors; 2) parental consent; 3) balancing benefits and risks; and 4) data protection and release. These key points were developed to guide decision-making regarding pediatric data sharing (Rahimzadeh et al., 2018).

While policy is essential to fostering a data sharing culture, practical tools are required to turn policy into action. Consent clauses are examples of such tools. Subject to local laws and disease-specific adaptation, model consent clauses can facilitate and promote data sharing and research, while ensuring compliance with ethical and legal norms (Nguyen et al., 2019). Indeed, this has already been demonstrated in the case of data sharing for research on rare diseases (Nguyen et al., 2019). In this paper, we discuss how specific pediatric-related consent clauses may serve to facilitate data sharing while ensuring the appropriate safeguarding of pediatric data. We follow up on previous discussions in the literature by outlining how these clauses may be used as part of informed consent processes for the participation of minors in research. Tailoring the informed consent process to address the ethical issues raised by pediatric data sharing can thereby help facilitate the ethical and responsible sharing of pediatric data.

## Rationale for Pediatric Data Sharing

As previously stated, we argue that unduly restrictive data sharing policies should not unnecessarily impede potential future health benefits for children. Limited data sharing severely restricts scientific advancements, creating knowledge gaps which may negatively affect future generations (Rahimzadeh et al., 2017). International human rights have been argued as an ethical basis for increased data sharing (Knoppers & Joly, 2007; Knoppers et al., 2014; Rahimzadeh et al., 2017; Rahimzadeh et al., 2018). For instance, under the United Nations *Convention on the Rights of the Child* (United Nations General Assembly, 1989)*,* the best interests of the child are primary (art. 3) and children have both the right to be heard (art. 12) and the right to the highest attainable standard of health (art. 24). Pediatric data sharing should be understood as an extension of these principles and of the human right of everyone to benefit from scientific advancements (United Nations General Assembly, 1948, art. 27; Knoppers et al., 2014; Knoppers & Joly, 2007; Rahimzadeh et al., 2017).

Pediatric longitudinal studies, which generate rich datasets, only reinforce the need for greater data sharing. Consortia-based genomic research has greatly changed the ways in which data are collected, stored, and shared for research purposes, including in pediatric research (Rahimzadeh et al., 2017). This is especially important in the pediatric context. Childhood diseases are often rare and heterogeneous (Bennett et al., 2014; Bennett et al., 2019; Vaske & Haussler, 2019). Single-centre pediatric studies often lack sufficient sample sizes to produce meaningful research results (Bennett et al., 2019). Pediatric data sharing is therefore key to filling potential lacunae in research (Bennett et al., 2019). This may not only translate into benefits for the minor to whom the data relates (e.g., the development of specific treatments), but also to future generations of minors who may benefit from the results of the research. Indeed, research ethics guidelines recognize that, in certain cases where the research does not entail any direct benefit to the participants, it should have the prospect of providing benefits to other minors who stand to benefit from the research (e.g., World Medical Association, 2013; Canadian Institutes of Health Research et al., 2018).

One example of consortia-based pediatric research is the Developmental Research in Environmental Adversity, Mental health, Biological Susceptibility and Gender (DREAM-BIG). DREAM-BIG brings together three longitudinal pregnancy cohorts: Avon Longitudinal Study of Parents and Children (UK), The Generation R Study (Netherlands) and Maternal Adversity Vulnerability and Neurodevelopment (Canada) (Szekely et al., 2021). This collaborative research initiative seeks to understand the effects of prenatal maternal adversity on child psychopathology (Szekely et al., 2021). The ability to share data between cohorts internationally is critical to ensuring sufficient statistical power to produce generalizable knowledge (Beauvais et al., 2021).

## Consent Issues in Pediatric Genomic Research

Informed consent is the cornerstone of the ethical conduct of research. With the move towards increased data sharing, informed consent is required not only for participation in research, but for data sharing as well. In many jurisdictions, consent is required for data sharing, which has often been highlighted as a legal barrier, especially where data is shared across jurisdictional boundaries (Poline et al., 2012; Kosseim et al., 2014; Wiebe & Dietrich, 2017). For this reason, informed consent processes should be tailored to recognize and notify participants of the data sharing context. Moreover, where the data is being collected from minors, additional consent issues need to be addressed. For one, minors generally lack the legal capacity to provide informed consent and, instead, their parents (or legal guardians) provide informed consent on their behalf (Hens et al., 2013; Varadan, 2020). Minors may, however, indicate their agreement to participate by providing their assent, if they are able to understand the significance of the research (Dalpé et al., 2019). Nevertheless, some jurisdictions recognize the legal capacity of “mature minors” to consent independently to research, based on the individual professional determination of their ability to fully understand the research and its implications. Other jurisdictions have a set age of presumed “medical” maturity before the age of legal capacity through specific legislation (Knoppers et al., 2016). Accordingly, pediatric consent clauses should be tailored to both mature minors and parents (or legal guardians).

Furthermore, informed consent processes need to consider the specific issues raised by pediatric research and the sharing of pediatric data. Data confidentiality and the privacy rights of minors, for one, are critically important and data safeguards need to be well outlined and explained to parents (or legal guardians). Moreover, where data and samples are collected from minors, there arises the issue of obtaining permission to continue to store and share the data when the minor reaches the age of majority or maturity. In the following sections, we discuss how some of the key components of the GA4GH pediatric consent typology (Global Alliance for Genomics & Health, 2021) can help address the key issues of pediatric data sharing, including many of the policy points raised in the KIDS Framework (Rahimzadeh et al., 2018). These consent clauses were developed by GA4GH to address the specificities of pediatric research, including data sharing and are based on different consent templates used around the world. Leveraging these consent clauses can help facilitate data sharing in a responsible manner, reconciling the benefits of data sharing with protecting participants’ rights and interests.

## Discussion: Typology of Model Pediatric Consent Clauses

### Assent

Minors who are not mature or who are not considered by law to have medical maturity, should be given the opportunity to provide their assent. While there is no formally agreed-upon definition of assent (Giesbertz et al., 2014; Varadan, 2020), it is generally considered an expression of the willingness of a minor to participate in the research when they are capable of understanding the general purpose of the research (Dalpé et al., 2019). Indeed, one of the key policy points of the KIDS Framework is the involvement of minors in all data sharing-related decision-making in an age-appropriate manner (Rahimzadeh et al., 2018).

Where minors are enrolled in research, the informed consent process should be understood as encompassing both parental (or legal guardian) consent and the minor’s assent, where applicable (Rotz & Kodish, 2018). While they are generally unable to understand how they’re data will be shared and used, it is good practice to provide minors with age-appropriate information about how their data will be shared and used (Hens et al., 2013). Moreover, studies indicate that minors want to be involved in such decision-making processes (Giesbertz et al., 2014). Consent forms should therefore include assent language that is clear and comprehensible to the minor. Researchers should also consider obtaining and noting verbal assent if more appropriate to the minor’s level of maturity. Including assent clauses (or obtaining verbal assent) therefore acknowledges the agency of minors and their right to be heard (Rahimzadeh et al., 2018). While there may be certain exceptions, a minor’s expression of disagreement (called dissent) should preclude their participation in the research (Ross, 2006).

### Confidentiality

As in all forms of biomedical research involving human beings, researchers have a duty to safeguard the confidentiality of research participants’ personal information. Minors have a right to personal privacy and to the protection of their medical and other information (Beauvais & Knoppers, 2021). Yet, they may not fully comprehend the current and potential future re-identification risks related to genetic or genomic information. This inability to adequately appreciate these risks underpins the characterization of minors as vulnerable research participants. Adequate ethical processes and legal safeguards are required, and mature minors and parents should be informed of how the confidentiality of their information will be protected and what safeguards will be in place (Rahimzadeh et al., 2018).

With data sharing becoming increasingly commonplace in the scientific community, especially open data sharing, the importance of maintain strict safeguards cannot be overstated. This is all the more important where pediatric data is concerned, especially genomic data which may be characterized as inherently identifiable information (Joly et al., 2016). Moreover, there have been recent initiatives in the scientific community towards making data more accessible, such as through open access platforms (Joly et al., 2016). Studies have shown that parents make more restrictive decisions concerning the sharing of their children’s data than adults making decisions concerning their own data, citing future unknown risks to their children (Burstein et al., 2014). Moreover, parents have been shown to value the protection of privacy more than advancing scientific research (Burstein et al., 2014). This may explain, in part, parental concerns over potential misuses of their children’s data and their restrictive decision-making regarding the sharing of their children’s data (Barassi, 2020).

An appropriate balance must be achieved between protecting confidentiality and privacy rights and sharing data to achieve positive research outcomes (Wright et al., 2018). Where parents are consenting on behalf of a minor, confidentiality safeguards need to be properly outlined to address these concerns and to promote informed decision-making. Indeed, researchers are responsible for discussing potential risks (as well as benefits) to parents during the informed consent discussions (Rahimzadeh et al., 2018).

### Broad Consent for Research Use

Broad consent clauses for actual and future approved data sharing should be included in consent forms for pediatric research. Though there is disagreement in the literature over the scope of broad consent, it is generally defined as consent for future unspecified research uses, subject to ethics review and oversight (Hens et al., 2013; Riggs et al., 2019; Sanderson et al., 2017). Limiting consent to single research uses or to specific jurisdictions limits the ability to share data. Moreover, allowing data to be shared and used for future unspecified research uses helps minimize duplicative data collection (Poline et al., 2012).

Yet, mature minors and parents should also be informed upon joining a research study of how their data will be shared, whether in coded form (i.e., direct identifiers are removed from the data and replaced with a code) or in anonymized form (i.e. irretrievably delinked) in open access platforms. One of the key components of the KIDS Framework is that anonymized data be made widely available through publicly accessible databases, whereas identifiable data be made available in coded form through controlled or registered access processes (Rahimzadeh et al., 2018). In all cases, mature minors and parents should be provided with information regarding the governance and oversight of the data, of where, for how long, and how the data will be securely stored, as well as what access governance processes will be implemented for data sharing. Indeed, parental consent for future unspecified research uses of minors’ data should include information related to data governance practices (Rahimzadeh et al., 2018).

### Recontact

It is a general ethical requirement that when minors enrolled in research later become able to provide informed consent, they should, if possible, be recontacted to be asked to provide their own informed consent (Knoppers et al., 2016; Christensen et al., 2017; Giesbertz et al., 2016; Hens et al., 2013). Recontact is premised upon the ethical principle of respect for autonomy, allowing the now-adult or mature research participant to make their decisions about how their data or samples should be used (Giesbertz et al., 2016). Moreover, recontact may also be based on respect for the minor’s right to an open future, by providing them the option of either consenting to the continued storage and sharing of their data, or withdrawing consent altogether (Goldenberg et al., 2009; Beauvais & Knoppers, 2021). Where researchers intend to continue to use participants’ data or samples after they acquire the legal age of capacity or maturity, they should therefore recontact participants and ask them to provide their own consent to the continued storage and sharing of their data and samples. Consent may be obtained either through a formally re-consenting the participant, or simply by notifying them of their participation and of their right to withdraw (notification with opt-out) (Knoppers et al., 2016; Patrinos et al., 2022). In certain cases, waivers of recontact may be sought if certain conditions are met (Knoppers et al., 2016; Patrinos et al., 2022).

Mature minors and parents should be asked if they wish to be contacted for potential future participation in other research projects or to provide additional data and samples in the existing study. This can help create future opportunities for the collection of new or expanded datasets for sharing. Even if they agree to be contacted, mature minors and parents should be informed that they remain free at all times to decide whether or not they wish to participate in these other studies when asked in the future or to provide additional data or samples. Thus, permission to recontact should ideally be in the initial consent.

### Withdrawal

It is a basic principle of research ethics that participants are free to withdraw from the research at any point without any reason (World Medical Association, 2013). Minors should be also free to withdraw their assent at any time without providing a reason. Moreover, parents are free to withdraw their children from research. Also, as discussed above, upon recontact at the age of majority (or maturity), minors have a right to withdraw their data (Giesbertz et al., 2016; Hens et al., 2013; Knoppers et al., 2016; Christensen et al., 2017). Researchers should therefore outline how participants’ data will be managed upon withdrawal, including whether the data will be stored but no longer used or whether it will be destroyed. At the time of the initial consent and upon recontact, minors and parents should be informed that it may not be possible to retrieve data that has already been shared (Melham et al., 2014). Mature minors and parents should also be informed of any limitations related to withdrawal. For instance, anonymized data cannot be withdrawn, since it is not possible to re-link the data with the participant. Moreover, data which have been shared and used by other researchers in their work and publications or presentations cannot be retrieved. These limitations should be specified and explained to participants to promote informed decision-making.

## Conclusion

Biomedical research has become an increasingly data-intensive and collaborative venture. The sharing of data, including genomic and health-related data, is key to advancing research and is now a widely recognized ethical and scientific imperative. However, minors are very often excluded from data sharing initiatives due to various ethical issues, such as the sensitivity of pediatric genomic data, lack of capacity to provide informed consent, and potential re-identification risks. Overly protectionist policies have impeded data sharing initiatives which may otherwise lead to important health benefits for both current and future generations of minors. An optimal balance must be achieved between facilitating pediatric data sharing (and its associated positive outcomes) and ensuring that appropriate safeguards are in place to address the actual and future ethical issues raised by the sharing of pediatric data.

Previous literature has examined developing policy considerations to promote the ethical and responsible sharing of pediatric data (Rahimzadeh et al., 2018). This article follows up on these previous initiatives by outlining how consent clauses tailored to the pediatric data sharing context can help translate policy into practice. Using the pediatric consent clauses developed by the GA4GH’s as a case in point, we have illustrated how the ethical issues raised by pediatric data sharing can be addressed during informed consent discussions. Together, these model consent clauses can be leveraged to not only promote, but facilitate pediatric data sharing.

## Data Availability

The original contributions presented in the study are included in the article/supplementary material, further inquiries can be directed to the corresponding author.
